# Stimulation Parameters Used During Repetitive Transcranial Magnetic Stimulation for Motor Recovery and Corticospinal Excitability Modulation in SCI: A Scoping Review

**DOI:** 10.3389/fnhum.2022.800349

**Published:** 2022-04-07

**Authors:** Nabila Brihmat, Didier Allexandre, Soha Saleh, Jian Zhong, Guang H. Yue, Gail F. Forrest

**Affiliations:** ^1^Tim and Caroline Reynolds Center for Spinal Stimulation, Kessler Foundation, West Orange, NJ, United States; ^2^Department of Physical Medicine and Rehabilitation, Rutgers—New Jersey Medical School, Newark, NJ, United States; ^3^Center for Mobility and Rehabilitation Engineering Research, Kessler Foundation, West Orange, NJ, United States; ^4^Burke Neurological Institute and Feil Family Brain and Mind Research Institute, Weill Cornell Medicine, White Plains, NY, United States

**Keywords:** neuromodulation, recovery, stimulation parameters, plasticity, variability, spasticity

## Abstract

There is a growing interest in non-invasive stimulation interventions as treatment strategies to improve functional outcomes and recovery after spinal cord injury (SCI). Repetitive transcranial magnetic stimulation (rTMS) is a neuromodulatory intervention which has the potential to reinforce the residual spinal and supraspinal pathways and induce plasticity. Recent reviews have highlighted the therapeutic potential and the beneficial effects of rTMS on motor function, spasticity, and corticospinal excitability modulation in SCI individuals. For this scoping review, we focus on the stimulation parameters used in 20 rTMS protocols. We extracted the rTMS parameters from 16 published rTMS studies involving SCI individuals and were able to infer preliminary associations between specific parameters and the effects observed. Future investigations will need to consider timing, intervention duration and dosage (in terms of number of sessions and number of pulses) that may depend on the stage, the level, and the severity of the injury. There is a need for more real vs. sham rTMS studies, reporting similar designs with sufficient information for replication, to achieve a significant level of evidence regarding the use of rTMS in SCI.

## Introduction

Spinal Cord Injury (SCI) is defined as a traumatic or non-traumatic event affecting the spinal cord that results in sensory, motor, and autonomic deficits reducing independence and quality of life (QOL). In 2020, the National Spinal Cord Injury Statistical Center reported 294,000 people currently living with SCI (National Spinal Cord Injury Statistical C, 2020). Worldwide, this represents 2–3 million people, predominantly young adults, living with SCI related disability (Quadri et al., 2020). Over the last decade, due to advancements in medical procedures and patient care, survival rates after an SCI have increased (Alizadeh et al., 2019) and the length of acute stage hospitalization has dropped to 11 days as compared to 24 days in the 1970s (National Spinal Cord Injury Statistical C, 2020).

Improved understanding of the pathophysiological mechanisms underlying recovery after SCI have opened new perspectives for rehabilitation (Witiw and Fehlings, 2015; Fouad et al., 2020). Limited spontaneous motor function recovery after incomplete and complete lesions is at least partially due to cerebral and spinal plasticity processes involving spared and damaged circuitry (Raineteau and Schwab, 2001; Fink and Cafferty, 2016). At 1 year post-injury, 70% of cervical complete SCI individuals recovered one motor level, but only 30% recovered two or more motor levels (Steeves et al., 2011). The recovery rate is lower after complete compared to incomplete SCI (Ditunno et al., 2000). Most injured individuals remain burdened with significant SCI-related deficits.

SCI interrupts the connection between the brain and the body periphery; to restore lost functions, new connections need to be made, which necessarily involves axon growth and synaptogenesis. In rodent studies, actual axonal sprouting, and corticospinal tract (CST) regeneration has been shown following a lesion (Liu et al., 2010; O’Donovan et al., 2014). Regeneration can be promoted by existing neuromodulatory interventions such as high frequency repetitive transcranial magnetic stimulation (HF-rTMS). Indeed, studies have shown that HF-rTMS can increase the levels of brain derived neurotrophic molecule (BDNF) in rats’ central nervous system (Gao et al., 2017; Fujiki et al., 2020). This increase is thought to reflect mechanisms of structural and synaptic plasticity (Bliss and Cooke, 2011).

rTMS is a non-invasive brain stimulation (NIBS) technique that relies on the principle of electromagnetic induction of Faraday. A rapidly changing magnetic field in the TMS coil induces a brief electric current in the brain which generates secondary currents responsible for spreading neuronal activation at the cortical and subcortical levels (Barker et al., 1985; Lefaucheur, 2019). The underlying effects are thought to be mediated by long-term potentiation (LTP) and depression (LTD) -like mechanisms. The repeated administration of the magnetic pulses, at a certain frequency, are thought to induce short- to long-term changes in corticospinal excitability (CSE) and affect plasticity mechanisms. Until recently, the frequency of stimulation was thought to be the main determinant of the after-effects, with low frequency rTMS (LF-rTMS, <1 Hz) inducing a decrease of CSE whereas HF-rTMS (≥5 Hz) induces its increase (Rossi et al., 2009, 2020).

A recent review evaluated real vs. sham rTMS protocols, covering decades of research on therapeutic rTMS efficacy for several neurological conditions including neuropathic pain, depression, and stroke (Lefaucheur et al., 2020). For SCI, this field is novel with limited published research. Preliminary results suggest potential benefits for motor and sensory recovery, as well as addressing secondary complications such as spasticity and chronic pain. Recent reviews have suggested that rTMS is a promising neuromodulatory therapeutic tool that may help recovery after SCI (Ellaway et al., 2014; Tazoe and Perez, 2015; Gunduz et al., 2017), however, there is still insufficient evidence supporting rTMS use in clinical settings. Moreover, standardized rTMS protocols defining optimal stimulation parameters (i.e., stimulation frequency, intensity, duration of trains, number of pulses, etc.), number of sessions and duration of each session, and potential combination with other rehabilitation interventions remain to be determined.

Outcome variability is a well-known issue in the rTMS field (Sale et al., 2007; López-Alonso et al., 2014; Schoisswohl et al., 2019; Xiang et al., 2019). In tinnitus (Schoisswohl et al., 2019), the authors addressed the problem using a reviewing methodology based on study frequency that helped them infer optimal stimulation parameters. In SCI, Leszczyńska et al. (2020) described the use of an algorithm to define individual stimulation parameters based on individual SCI participant response to TMS. The resulting individualized parameters were however not explicitly reported.

Thus, instead of addressing only the therapeutic potential of rTMS in SCI, a topic already covered in previous reviews with positive conclusions, our focus here is to describe and discuss rTMS protocol design, aiming to highlight stimulation parameters that are likely to induce beneficial effects on motor function recovery, spasticity, and/or CSE after SCI. We conclude by making some recommendations for future research studies involving SCI individuals.

## Methods

### Search Methodology and Study Selection

To identify the most relevant studies, a literature search in PubMed, MEDLINE (OVID) SCOPUS, and Cochrane Library databases was performed in the abstracts and/or titles using two general key concept words “spinal cord injury (SCI)” and “repetitive transcranial magnetic stimulation (rTMS)”. Articles studying the effect of rTMS intervention on upper- and lower-extremity motor function and deficits, spasticity, and CSE in SCI individuals were examined. In addition, studies related to pain and sensory deficits were considered when the *rTMS targeted the motor cortex* and reported *independent outcomes of CSE*. We included randomized controlled, as well as non-randomized, longitudinal trials and studies that investigated the effects of rTMS *when combined with other rehabilitation interventions* and *single-case reports*.

The exclusion criteria were studies focusing on effects of patterned rTMS stimulation interventions, [i.e., paired associative stimulation (PAS) or theta burst stimulation (TBS)] or other forms of NIBS (i.e., tDCS or electrical stimulation alone), and studies reporting rTMS effects on pain or sensory function exclusively.

All articles meeting the above inclusion/exclusion criteria published in English up to mid-January 2021 were included and reviewed. A gray literature search and reference lists of the selected articles were also scanned to identify potentially relevant sources and additional studies.

### Additional Exploratory Analysis

Given the small number of randomized controlled trials (RCT) in the rTMS-SCI field and the difficulty of calculating the effect size from the included studies, we chose to conduct an exploratory analysis based on the frequency of studies reporting significant or non-significant outcomes after the rTMS interventions (Schoisswohl et al., 2019). The frequency analysis was performed for a selection of rTMS parameters and characteristics, each of which was divided into subcategories. An excel table was completed with the data extracted from the included articles. The parameters analyzed (columns) were entered for each specific reviewed article (rows). Most of the subcategories (numerical categories: i.e., *number of sessions*, *number of pulses*…) were defined and subdivided based on a cutoff value corresponding to the median value calculated for a specific study parameter. Regarding “*stimulation frequency*” parameter, given that most of the included articles used HF protocols, we have chosen to set the cutoff value at 10 Hz to separate those which used commonly used frequencies (high: 5–10 Hz) from those which used less common and higher frequencies (very high: 15–20–22 Hz) of stimulation and thus also obtaining a similar number of studies in both sub-categories. Two main categories of outcomes were defined, i.e., “clinical” (which includes measures related to motor deficits, spasticity, QOL, and activity of daily living, ADL) and “neurophysiological” (which includes only neuro-electrophysiological measures of CSE). A study was considered significant for a given effect category if two or more of the used outcomes of the main outcome category were reported as significant.

## Results

### Description of Included Studies

**Figure 1:**
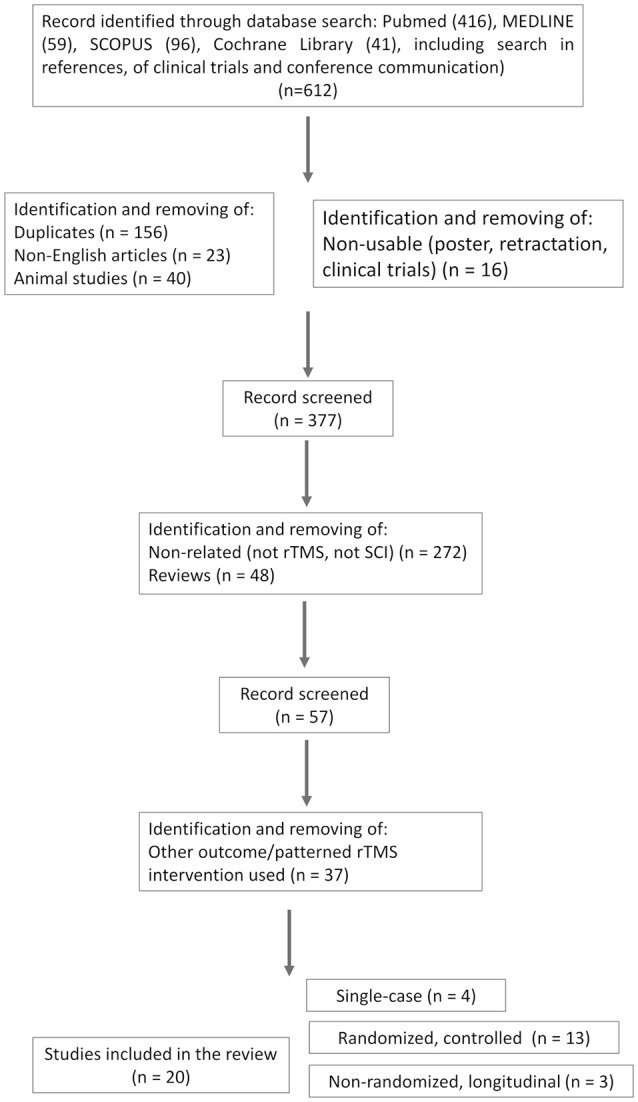
Study selection flow diagram, in accordance with PRISMA-ScR guidelines.

This study follows the Preferred Reporting Items for Systematic Reviews and Meta-Analyses (PRISMA) Extension for Scoping Reviews (PRISMA-ScR) guidelines (Tricco et al., 2018; see flow diagram in Figure 1). The search strategy resulted in a total of 612 records (416 articles in PubMed, 59 in MEDLINE, 96 in SCOPUS, and 41 trials in the Cochrane library). A first step was to identify and remove duplicates (*n* = 156), as well as non-English (*n* = 23), animal (*n* = 40), and non-exploitable studies (such as poster, clinical trials design without sufficient information about protocol designs, and retracted studies, *n* = 16). Non-related studies (i.e., not using rTMS or focusing on other pathology, *n* = 272) and reviews (*n* = 48) were removed. The remaining articles (*n* = 57) were screened more carefully against the inclusion/exclusion (I/E) criteria. Studies using other patterned rTMS intervention (i.e., PAS, TBS) or those testing rTMS for other purposes (pain alone; *n* = 37) were excluded.

Thus, after full-text examination according to the I/E criteria, 20 articles were retained; four were single-case reports and three were one arm(s) longitudinal studies. The 13 remaining were randomized controlled studies, of which six were randomized double-blinded cross-over placebo-controlled [one is a published study protocol with enough information about the protocol design to be included (de Araújo et al., 2017), one randomized simple-blinded cross-over placebo-controlled study, and six randomized blinded parallel placebo-controlled studies]; 13 studies were combined with other rehabilitation interventions and seven studies were not. One study involved bilateral rTMS [right and left M1 successive stimulation during the same session (Leszczyńska et al., 2020)]. One tested two rTMS conditions (leg and hand motor areas stimulation) vs. sham (Jetté et al., 2013).

The demographic and clinical information extracted were the *number of participants* in the study, SCI individuals’ *deficit levels and severity* (as measured with the American Spinal Cord Injury Association (ASIA) impairment scale, AIS) and *time since injury* (in days, months, or years). Study-specific information included were the *study design*, the *associated intervention* (i.e., motor training, functional stimulation, robotic training), or the *control condition* (if present), and the *outcomes measures* used to assess to the intervention effects. The most common rTMS parameters identified and extracted were the *TMS device*, *coil type*, *targets of stimulation*, *TMS frequency* (in Hz), *TMS intensity* (%) and the *method/muscle used to find the threshold*, the *number of pulses*, the *number of bursts per session* and its *duration*, the *number of total pulses*, the *duration of session*, the *number of sessions* and the *inter-trains interval (ITI)*. The pulse waveform and coil orientation were not reported explicitly and systematically in all studies and are therefore not included in the review. *Neuronavigation* was considered not used when not explicitly reported. Table 1 summarizes specific information from all included articles on the population studied, the rTMS intervention design (i.e., duration, frequency, …etc.), the outcome measures and side effects.

**Table 1:** Descriptive table of the reviewed rTMS studies.

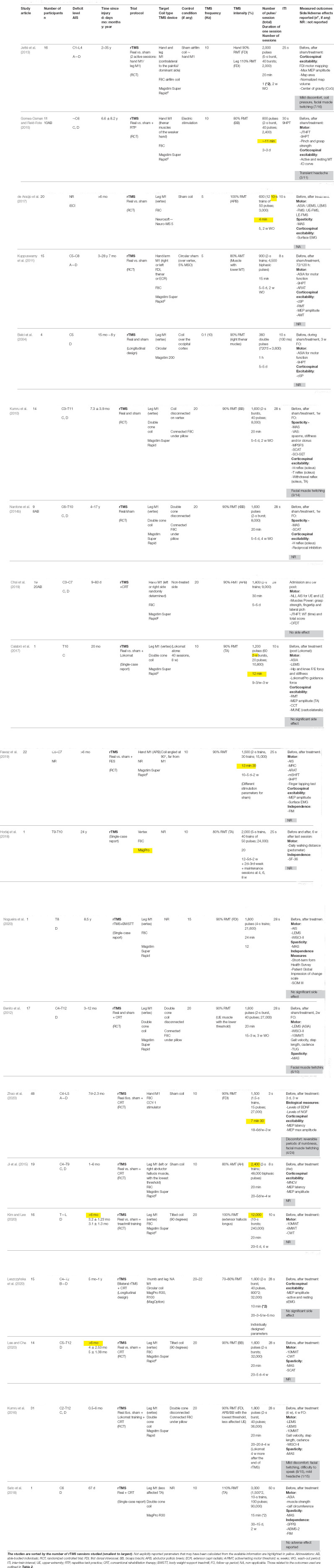

***The studies are sorted by the number of rTMS sessions studied (smallest to largest)**. Not explicitly reported parameters that may have been calculated from the available information are highlighted in yellow. Abbreviations: AB, able-bodied individuals; RCT, randomized controlled trial; FDI, first dorsal interossei; BB, biceps brachi; APB, abductor pollicis brevis; ECR, extensor capri radialis; A/RMT, active/resting motor threshold; w, weeks; WO, wash-out period; ITI, inter-train interval; UE, upper-extremity; RTP, repetitive task practice; CRT, conventional rehabilitation therapy; BWSTT, body weight-support treadmill; FO, follow-up period; NA, non-applicable. Those related to the outcomes used are outlined in Table 2*.

### Outcome Measures Used to Assess the rTMS Effects in SCI

Table 2 lists and describes the clinical and functional outcomes, as well as the spasticity and QOL/ADL measures, used in at least two studies.

**Table 2:** List and description of the outcomes and measures used in the rTMS studies.

	**Description and scoring**	**ICF Domain/ measurement domain**
**Clinical measures**		
American spinal injury association (ASIA) scale (AIS)	Level and severity of the injury (A to D; ISNCSCI 2019 - American Spinal Injury Association, 2019) UE and LE measures: no contraction (0) to normal resistance (5) UEMS: /50 LEMS: /50	Body function Motor and Sensory
Walking index for spinal cord injury (WISCI, WISCI-II)	Walking independence, functional mobility, and walking. Type, amount of assistance and device needed (Ditunno et al., 2013). Scores: unable to walk (0) to independent walking (20)	Activity Motor
***Upper-extremity function***		
Nine-hole pegboard test (9HPT, NHPT)	Finger dexterity (Huertas-Hoyas et al., 2020). Time taken to complete the test activity (s) Number of pegs placed during 50 or 100 s.	Body function, activity Motor
Jebsen-Taylor hand function test (JTHFT)	Fine and gross motor hand function using simulated ADL (Huertas-Hoyas et al., 2020) Time taken to complete the test (s)	Participation Motor
Action Research Arm Test (ARAT)	Upper extremity performance (coordination, dexterity and functioning; Hsieh et al., 1998). A 4-point ordinal scale: (0) to maximum and better performance (57)	Activity Motor
Pinch, grasp strength test	Measure the maximum isometric strength of the hand and forearm muscles when doing a pinching/grasping action Testing is repeated 3 times and an average is calculated (kg, lbs)	
***Lower-extremity function***		
10-meter walk test (10-MWAT)	Functional mobility, gait and vestibular function (Amatachaya et al., 2014) Gait speed (m/s) during 10 meters walk.	Activity Motor
Community walk test (CWT)	LE functioning and mobility Time to walk 300 m in the community with no (1) or quadruped cane (6) aid	Activity Motor
*Short physical performance battery (SPPB)*	Balance, lower extremity strength, and functional capacity (Ronai and Gallo, 2019) 3 items: no (0) to maximum (12) Balance, gait speed test, chair stand test	Activity
***Spasticity***		
Modified Ashworth scale (MAS)	UE and LE spasticity and resistance to passive movement of a joint with varying degrees of velocity (Pandyan et al., 1999). No increase of muscle tone (0) to rigid parts in flexion or extension (4)	Body structure and function Motor
Spinal Cord Assessment Tool for Spastic reflexes (SCAT)	Spastic LE behavior (Akpinar et al., 2017) Clonus (0, no reaction to 3, severe lasting >10 s) Flexor spasms (0, no reaction to 4, severe with >30° of hip and knee flexion) Extensor spasms (0, no reaction to 4, severe with >30° of hip and knee flexion)	Body structure and function Motor
***Quality of life (QOL) and daily living (DL)***		
*Short-term form Health Survey (SF-36)*	Health status in the Medical Outcomes Study (Ware and Sherbourne, 1992) 36 questions, 8 domains of health Total score indicating a range of low to high QOL	Participation Quality of life
*Patient Global Impression of change scale (PGICS)*	All aspects of patients’ health: improvement or decline in clinical status. no change (1) to considerable improvement (7)	ADL, quality of life
*Functional independence measures (FIM)*	level of patients’ disability (level and function) and amount of assistance needed to carry out ADL (Sivan et al., 2011) 13 motor tasks and 5 cognitive tasks Independence: complete dependence (0) to complete independence (7) Level of function: lowest (18) to highest (126)	Activity ADL, motor, cognition
**Corticospinal excitability**		
*RMT/AMT*	Excitability of the central core of the corticomotor neurons and their membrane excitability (Nardone et al., 2015). %MSO	
*MEP*	Corticospinal excitability, cortico-muscular conduction (Nardone et al., 2015) At rest or during active muscle contraction Mean/max amplitude or area, latency	
Hoffmann reflex (H-reflex)	Spinal excitability. Modulation of monosynaptic reflex activity in the spinal cord (Knikou, 2008).	

*Abbreviations: U/L EMS, upper/lower-extremity motor scores; QOL, quality of life; R/A MT, resting/active motor threshold; MSO, maximum stimulator output; MEP, motor evoked potential*.

#### Biological Substrates and Side/Adverse Effects

Other relevant outcomes include the *blood levels of BDNF (Brain-derived neurotrophic factor) and NGF (nerve growth factor)*. BDNF and NGF levels reflect rTMS effects on structural plasticity mechanisms (i.e., axon regeneration; Bliss and Cooke, 2011; Moxon et al., 2014). *Side effects (SE) and adverse events* (AE) related to TMS administration were also reported.

### Main Observed Effects of rTMS in SCI

#### CSE Changes Associated With Analgesic Effects

One study applied a *single-session of HF-rTMS* tochronic SCI participants and included measures of pain and CSE (Jetté et al., 2013). The analgesic effects observed were associated with increase of CSE, as demonstrated with an increase of MEP amplitude. Similar effects on pain and CSE were observed in subacute SCI patients after repeated HF-rTMS over *18 sessions*, associated with an increase of biological markers levels (BDNF and NGF; Zhao et al., 2020). The other stimulation parameters (*frequency of 10 Hz, subthreshold stimulation intensity*) were similar in both studies.

#### Upper-Extremity Function and Associated CSE Changes

Modest to good and maintained effects on UE function and CSE seem to be associated with rTMS. Gomes-Osman and collaborators tested the effects of *three sessions* of HF-rTMS over the *hand M1*, *associated with repetitive task practice (RTP)* during the inter-train interval in 11 chronic iSCI (Gomes-Osman and Field-Fote, 2015). They showed improvement in grasp strength and the ability to perform the JTHFT (as showed with higher effect size in the active group) beyond those observed with RTP alone, with inter-manual transfer of the training effects observed. No changes in CSE were observed. Using a similar protocol but *repeated over 5 days (rather than 3)* and *without using any additional hand training* in 15 chronic SCI, Kuppuswamy et al. (2011) observed only modest, not maintained functional gain in ARAT score and increase in FDI’ AMT at 72 and 120 h post-rTMS intervention. Both studies reported no between-condition (active vs. sham) difference in hand motor performance or CSE. Belci et al. (2004) were the first to test and administer a rTMS protocol to four SCI patients in the chronic stage, repeated over 5 days (Belci et al., 2004). They used a specific design of *high frequency double pulses* administered at low-frequency (0.1 Hz), at an intensity of 90% RMT for 5 days over the leg motor area *(leg M1, vertex)*. They demonstrated beneficial effects on motor and sensory function and dexterity, using the AIS and 9HPT respectively, and reduced CS inhibition and electrical perceptual threshold. The clinical changes were maintained for weeks whereas the electrophysiological changes returned to pretreatment levels at follow-up. Choi et al. (2019) specifically tested five *sessions* of HF-rTMS *(20 Hz)* in central cord syndrome patients, the most common type of SCI (Choi et al., 2019). They reported improved motor function with increased JTFHT time and scores and muscle strength thus demonstrating once again the potential of HF-rTMS to improve fine motor performance. With *10 sessions* of *10 Hz-rTMS associated with manual training program*, Fawaz et al. (2019) reported improvements in overall UE deficits and motor function associated with CSE increase.

#### Spasticity, Lower-Extremity Function, and CSE

The effect of *daily stimulation sessions over 1 week* on spasticity symptoms was explored. A modulation and improvement of knee spasticity symptoms were demonstrated after *20 Hz-rTMS* over the *leg M1* on 15 iSCI participants (Kumru et al., 2010). These changes were maintained at 1 week follow-up but were, however, not associated with neurophysiologic changes in iSCI. The same protocol was tested in 2014 in nine SCI at a more chronic stage of their injury (Nardone et al., 2014a). Real rTMS was observed to significantly reduce LE spasticity (decrease of MAS and SCAT scores), associated this time with neurophysiological effects, as reflected by a decrease of reciprocal inhibition. Both studies reported beneficial effects from the first session of the proposed rTMS protocol.

The effects on LE function and spasticity seem more significant and maintained when HF-rTMS is *associated with other rehabilitation interventions*. Improvements in LE muscle strength, spasticity, and gait were demonstrated following *15 sessions* of HF-rTMS at *20 Hz associated with rehabilitative training* in 17 iSCI participants (Benito et al., 2012). These improvements were maintained 2 weeks after the protocol ended. In 2016, Kumru tested the same protocol as Kumru et al. (2010) and Nardone et al. (2014a) in more severe and subacute patients, using *20 sessions* of *20 Hz-rTMS* protocol and *associated with a robotic treadmill training (Lokomat)* (Kumru et al., 2016). They observed significant improvement in limbs motor scores. These improvements were greater in the real compared to the sham group and were maintained at 4 weeks follow-up for gait performance. Similarly, Calabrò et al. (2017) reported improvements in clinical scores, kinetic parameters, and CSE as an increase in MEP amplitude and MUNE in one chronic iSCI participant after combining HF-rTMS sessions with *Lokomat gait training*. Improvements in walking independence, functional mobility, and QOL, as measured with the WISCI-III, MAS, SCIM-III, and SF-36 respectively, were reported after *12 sessions* of *15 Hz-rTMS combined with BWSTT* in one SCI individual, 8.5 years after his injury (Nogueira et al., 2020). Definite beneficial effects of HF-rTMS on LE functions and spasticity were also confirmed in two randomized, placebo-controlled, and parallel trials (Kim and Lee, 2020; Lee and Cha, 2020) where significant clinical improvements were observed in chronic iSCI compared to sham group. Kim and collaborators study (Kim and Lee, 2020) study used a *higher intensity of stimulation (100% RMT)* and the *combination with treadmill training*.

#### Quality of Life and Side/Adverse Effects

Several studies were also interested in investigating HF-rTMS effects on ADL and QOL. Most of these were single-case studies (Hodaj et al., 2018; Sato et al., 2018; Nogueira et al., 2020) and reported beneficial effects with increases in SF-36 scores. A *high-frequency* and *intensity (10 Hz, 110% RMT)* protocol *associated with rehabilitative training* and *repeated over 30 sessions*, was demonstrated to be safe and to produce motor functional recovery in one subacute patient with incomplete, cervical injury (Sato et al., 2018). Fawaz et al. (2019) reported significant increases of FIM scores in 22 cervical and chronic SCI participants (Fawaz et al., 2019); increases reported to be significantly higher for the group for whom functional electrical stimulation (FES) was combined with real rTMS compared to the group where sham rTMS was used instead.

Globally, rTMS interventions were reported safe and well-tolerated by SCI participants with no serious and significant SE and AE. Only mild rTMS-related discomfort was reported (Table 1, 6 over 20 studies). The most common SE were facial muscle twitching during the real rTMS sessions (Kumru et al., 2010, 2016; Benito et al., 2012; Jetté et al., 2013; Zhao et al., 2020) and transient headaches (Gomes-Osman and Field-Fote, 2015; Kumru et al., 2016). These side effects were reported in a small number of participants.

### rTMS Protocols Parameters Description

Each study used a specific combination of parameters for its rTMS intervention. The protocol designs are reported in Table 1. Some parameters were not explicitly reported (e.g., session duration), we thus reported and highlighted in yellow in Table 1 the parameters that could be inferred or calculated from the available information. The frequency analysis in Table 3 was performed for a selection of 10 rTMS protocol parameters and characteristics, each of which was divided into two or three subcategories. Among the 20 total included studies (N total), the single-case studies (3) (Calabrò et al., 2017; Hodaj et al., 2018; Sato et al., 2018; Nogueira et al., 2020) and the published clinical trial (1) (de Araújo et al., 2017) have not been considered. The study from Jetté et al. (2013) investigating both the leg and hand conditions separately was counted twice, bringing the number of studies included in the frequency analysis to 16 (N studies included). Eleven of the 16 studies assessed the clinical and/or neurophysiological effects of the intervention (assessed) and five did not assess either of the effects (not assessed; first row, Table 3). The same rationale was used for counting the number of relevant studies in all the subcategories. The study frequency calculation in each subcategory was described above (Methods—additional analysis). The results are presented in Table 3. The main purpose of this exploratory analysis is to clarify what has been done (or less done) in this emerging research field and to try to summarize the main results, according to the rTMS parameter used, which are discussed in more details in the section below.

**Table 3:** Study frequency table.

	**Clinical Effect**					**Neurophysiological effect**		
	**N total (%)**	**N studies included**	**Assessed not assessed**	**Significant (%)**	**Not significant (%)**	**Assessed/not assessed**	**Significant (%)**	**Not significant (%)**
Overall	20	16	11/5	9 (82)	2 (18)	11/5	5 (45)	6 (55)
Number of sessions								
1–5 (<1 week)	8 (40)	8	6/2	5 (83)	1 (17)	7/1	2 (29)	5 (71)
9–30 (≥1 week)	12 (60)	8	5/3	4 (80)	1 (20)	4/4	3 (75)	1 (25)
Session duration								
<20 min	6 (30)	4	3/1	2 (67)	1 (33)	4/0	3 (75)	1 (25)
≥20 min	14 (70)	12	8/4	7 (88)	1 (22)	7/5	4 (57)	3 (43)
Number of Pulses								
<1,600	6 (30)	5	4/1	3 (75)	1 (25)	5/0	2 (40)	3 (60)
≥1,600	14 (70)	11	7/4	6 (86)	1 (14)	6/5	3 (50)	3 (50)
Inter-train interval								
<28 s	10 (50)	8	4/4	1 (25)	3 (75)	7/1	4 (57)	3 (43)
≥28 s	10 (50)	8	7/1	6 (86)	1 (14)	4/0	1 (25)	3 (75)
Stimulation Frequency								
HF (≤10 Hz)	11 (55)	8	4/4	3 (75)	1 (25)	8/0	4 (50)	4 (50)
vHF (>10 Hz)	9 (45)	8	7/1	6 (86)	1 (14)	3/5	1 (33)	2 (77)
Stimulation Intensity								
Below (<100% MT)	17 (85)	14	9/5	8 (89)	1 (21)	10/4	5 (50)	5 (50)
At/above (≥100% MT)	3 (15)	2	1/1	1 (100)	0 (0)	1/1	0 (0)	1 (100)
Coil Type								
F8C	11 (55)	9	5/4	4 (83)	1 (17)	6/3	2 (33)	4 (67)
Circular	2 (10)	1	1/0	1 (100)	0 (0)	2/0	2 (100)	0 (0)
Double	6 (30)	4	4/0	3 (75)	1 (25)	2/2	0 (0)	2 (100)
NR	1 (5)	-	-	-	-	-	-	-
TMS device								
Magstim Super Rapid^2^	16 (80)	12	10/2	8 (80)	2 (20)	9/3	4 (44)	5 (56)
MagPro	3 (15)	1	0/1	-	-	1/0	1 (100)	0 (0)
CCY-1 Stimulator	1 (5)	1	0/1	-	-	1/0	0 (0)	1 (100)
Use of Neuronavigation								
No	18 (90)	13	11/2	9 (82)	2 (18)	8/5	4 (50)	4 (50)
Yes	2 (10)	3	0/3	na	na	3/0	1 (33)	2 (67)
Associated Rehabilitation								
No	8 (40)	6	4/2	3 (75)	1 (25)	6/0	2 (33)	4 (67)
Yes	12 (60)	10	7/3	6 (86)	1 (14)	5/5	3 (60)	2 (40)

*N total: total number of studies reviewed (*n* =20). N studies included: total number of studies where the parameters could be extracted and effects were described (the clinical trial and single-case studies were excluded and the study with two separated conditions was counted twice, *n* = 16); among which assessed or not the cited effect (clinical/neurophysiological). HF, high-frequency; vHF, very high frequency; MT, motor threshold; F8C, figure-of-eight coil*.

## Discussion

Our review confirms previous work about the seeming effectiveness of HF-rTMS to promote motor improvements and CSE changes in SCI. All the group studies reported significant improvements for at least one of the outcomes considered (cf Table 3). Interestingly, beneficial effects were reported in most studies despite the multiplicity and variability of the protocol designs used.

The observed effects of rTMS on sensorimotor function and spasticity in SCI individuals are thought to be due mainly to the rTMS-induced changes in CSE and CS connectivity (Gomes-Osman, Belci, Kumru, Sato) together with effects on cortical inhibition (Belci et al., 2004) resulting in alteration of spinal and supraspinal circuits and excitability (Nardone et al., 2014a). The excitability changes and plasticity-related phenomena are thought to be mediated through NMDA receptors (Rossini et al., 2015) and to involve several biological mechanisms such as synaptic plasticity (sprouting of new axons, guidance of axons to targets), remyelination, and spinal plasticity modulation as well as cell death limitation, cell regeneration, and replacement. Such effects are supported by increases in serum levels of neurotrophic factors such as BDNF and NGF (Min Hwang et al., 2014; Fujiki et al., 2020; Zhao et al., 2020).

Most rTMS studies in SCI showed promising and lasting functional gains, associated or not with neurophysiological changes (Table 3). However, **the relative novelty of the field in SCI and** the limited number of RCTs and the wide range of rTMS protocol design and parameters used precluded us, at this time, from performing a meta-analysis and drawing definite conclusions. An exploratory frequency analysis allowed us however to have some insights on the parameter settings that may maximize a particular symptom recovery.

The inter- and intra-individual variability in response to TMS and to NIBS, in general, is widely reported and studied in the literature (Sale et al., 2007; López-Alonso et al., 2014; Ovadia-Caro et al., 2019; Guerra et al., 2020). In able-bodied individuals, key influence factors were identified such as baseline MEP amplitude stimulus intensity and target muscle (Corp et al., 2020, 2021). It is likely that an inter- and intra-individual variability of NIBS response also exists in SCI, and that specific stimulation parameter changes can have a critical effect on the generated plasticity processes and the neurophysiological and/or clinical effects observed. In the next section, we will discuss the potential key source influencing the rTMS response in SCI, focusing first on the technical parameters and then describing other parameters, more related to the design of the rTMS and associated-rehabilitation sessions or to the participants themselves. We conclude by making some recommendations for the design and reporting of future rTMS studies in SCI.

### rTMS Technical Parameters

The **session duration** and the **number of sessions** are important factors to consider. Indeed, increased stimulation duration was shown to induce a more consistent increase in regional glucose metabolism and increase of neuronal activity in the stimulated area (Siebner et al., 2000; Thomson et al., 2011). Most studies (60% and 70%) used longer protocols (>1 week), with 20 min or higher duration per session. Increasing the number of sessions seems beneficial regarding both clinical and neurophysiological effects whereas, longer stimulation sessions had significant clinical effects (86%) with mixed results for neurophysiological effects (57%). However, few studies tested sessions shorter than 20 min (30%). Belci et al. (2004) used a 30 min rTMS protocol with significant and lasting effects on motor function and CS inhibition (Belci et al., 2004). One study out of the 20 reviewed reported the effect of single session of HF-rTMS on pain and CSE in chronic SCI (Jetté et al., 2013). Two others observed effects on spasticity and CSE after the first session of their repeated protocol (Kumru et al., 2010; Nardone et al., 2014a). No authors studied the effect of a single-session of HF-rTMS on motor function after SCI. Understandably, multiple sessions seem to be appropriate for maintaining the excitability and clinical effects (Benito et al., 2012; Kumru et al., 2016; Hodaj et al., 2018) and a higher number of training sessions with stimulation (≥1 week) is more likely to be associated with greater changes (Ji et al., 2015; Kim and Lee, 2020). However, quantifying the effect of a single session could provide important information on the mechanisms of effects obtained from multiple sessions.

The **number of pulses** and **duration of trains** delivered during a stimulation protocol is also critical to determine the after-effects of rTMS. Short trains were shown to decrease MEP whereas long trains increased MEP (Modugno et al., 2001; Peinemann et al., 2004). A small number of pulses (240) was also shown to produce less significant and consistent rTMS modulation effects in comparison with a larger number of pulses (1,600) (Maeda et al., 2000). The authors stated that 1,000 pulses or more might be needed to produce consistent rTMS effects. In SCI, three studies reported the use of a similar protocol in terms of daily stimulation pulses administration (720–900) and an overall number of sessions (3 to 4) on hand motor function (Belci et al., 2004; Kuppuswamy et al., 2011; Gomes-Osman and Field-Fote, 2015). Kuppuswamy et al. (2011) and Gomes-Osman and Field-Fote (2015) reported only modest changes compared to baseline, which were not different from sham intervention. In Kuppuswamy et al. (2011) the modest improvement might be also explained by the more heterogeneous study population in terms of lesion severity and time since injury. Among the reviewed articles, 70% of the studies used a higher number of pulses (>1,600 pulses) with mostly beneficial clinical (86%) and neurophysiological effects (Table 3). Four of them (Kumru et al., 2010, 2016; Benito et al., 2012; Nardone et al., 2014a) used a high number of pulse rTMS protocols (1,600–1,800) at 20 Hz during a 20 min intervention at 90% RMT and reported significant improvements in LE function and spasticity in SCI.

The rTMS after-effects depend on the interval between bursts of pulses, i.e., the **inter-train interval (ITI)**. It was shown that rTMS delivered continuously can be responsible for the reversal of the net effect from increased to decreased CSE (Rothkegel et al., 2010), explained by a hysteresis phenomenon and neuronal excitability saturation. The included studies used non-continuous stimulation with a wide range of ITI ranging from 3 s to 50 s. Those with an ITI ≥ 28 s seemed to more likely result in significant clinical effects (86% vs. 25% for ITI <28 s) while surprisingly the opposite is true for the neurophysiological effect. Shorter ITIs were shown to result in greater disinhibitory effects whereas longer breaks between trains might lead to a normalization of CSE due to increased cortical inhibition before the occurrence of the next burst reducing the effect summation of repeated bursts (Cash et al., 2017; Pitkänen et al., 2017).

Regarding **stimulation intensity**, only a few studies (*N* = 3) tested intensity at or above the threshold (Jetté et al., 2013; Sato et al., 2018; Kim and Lee, 2020). Most of the studies (85%) used an intensity below the threshold at 80%–90% R/AMT. Sato and collaborators used a protocol with 3,000 pulses delivered at an intensity of 110% RMT over 15 sessions and reported the safety and feasibility of such high-intensity, HF-rTMS protocol in one subacute SCI patient. The more intense stimulation would produce more enhancement of spinal longitudinal neurons, would stimulate broader cortical regions, and elicit faster temporal-spatial summation on corticospinal-motoneuron connections (Fitzgerald et al., 2002; Rossini et al., 2015). A parallel RCT confirmed the benefit of stimulating at higher intensity, with a significantly greater effect of real HF-rTMS administered at 100% RMT compared to sham on LE function of 16 iSCI participants at 6 months post-injury (Kim and Lee, 2020). The tested protocol also used a higher total number of pulses (12,000) at very high frequency (20 Hz) and combined with treadmill training. The benefit of using suprathreshold intensity needs to be further investigated. Leszczyńska et al. (2020) developed an algorithm to decide the optimal stimulation intensity based on SCI participants’ individual responses to TMS. Such individualized stimulation parameters may be an option to consider in the future; the procedure used to decide the parameters however needs to be detailed with accurate reporting of the results.

**Pulse frequency** was shown to be the major driver of the MEP change (Rodger et al., 2016). Also, high- (10 Hz; Dall’Agnol et al., 2014) but not low-frequency (1 Hz) rTMS was shown to increase BDNF levels (Mirowska-Guzel et al., 2013). This may explain why all the studies included in this review showed mostly beneficial effects. However, even if a wide range of high frequencies seems to provide consistent effects, the frequency-dependent increase in CSE due to rTMS at the group level was less clear at the individual level (Maeda et al., 2000). High- to very high-frequency rTMS (5–22 Hz) have generated both CSE and/or clinical beneficial effects in SCI individuals. However, to draw definite conclusions about the usefulness of rTMS in SCI, it may be worthwhile to also study the effects of LF-rTMS; especially given the possibly harmful hyperexcitability and increased inhibition that has been described after SCI (Petersen et al., 2012; Nardone et al., 2015) and that can be reversed by the administration of LF-rTMS protocols.

The **type of coil** influences the stimulated area. The double-cone coil seems to provide a deeper, stronger, and wider electric field (EF), but is also less focal compared to the one produced by a F8C (Lontis et al., 2006; Lefaucheur, 2019); which provides a deeper and more extensive magnetic field over the cerebral cortex than the usual circular coil (Lontis et al., 2006; Sato et al., 2018). A significant difference in MTs between F8C and double-cone coil for rTMS has been observed in patients with refractory depression, with systematically higher MT obtained with F8C (Miron et al., 2018). Targeting a wider area with a double-cone coil may be more appropriate for tetraplegic SCI patients. It is also important to keep in mind that when targeting the LE with a double-cone coil, one could also affect UE function (Kumru et al., 2016). In the literature surveyed here, F8C was the most used coil (55%) and the double cone coil was used in studies targeting LE and spasticity symptoms. Both were associated with mostly beneficial clinical effects in SCI. Modeling of the EF induced by different coil designs, achieved with newly developed tools (Saturnino et al., 2019; Aberra et al., 2020), may be useful to obtain additional information. Indeed, the head and EF modeling during brain stimulation can provide a better understanding of the rTMS underlying mechanisms, eventually, explaining the variability of the after-effects observed, and ultimately help to individually optimize the rTMS interventions (Konakanchi et al., 2020; Mosayebi-Samani et al., 2021). For example, it was demonstrated that the effect variability of a transcranial alternating current stimulation (tACS) intervention can be significantly predicted by measures derived from individual EF modeling (i.e., EF’ strength and spatial distribution; Kasten et al., 2019). It is reasonable to expect similar results with other NIBS such as rTMS, hence the need to include EF modeling in stimulation studies, especially those involving people with an injured central nervous system (Rossi et al., 2020; Mosayebi-Samani et al., 2021).

### Methods Used to Define and Assess rTMS Protocols

The **stimulation site**, or **hotspot of stimulation**, is a key factor that is chosen depending on the effect sought. The local neurophysiological changes and the associated clinical effects depend on the targeted cerebral area. It is important to keep in mind that when targeting the motor cortex (hand, arm, or leg M1), it is very likely to impact additional adjacent and remote connections and areas such as S1 (Belci et al., 2004; Kuppuswamy et al., 2011), due to the extended EF induced by the stimulation. This may explain the effects observed on sensory function (Belci et al., 2004) and the non-targeted side (Gomes-Osman and Field-Fote, 2015; Choi et al., 2019). Such bilateral and sensory effects may be of interest in SCI, given that stimulating the sensory cortex could have also benefit recovery (Pleger et al., 2006).

The rTMS intensity is often based on and defined at a specified percentage level of the **participant’s motor threshold** (e.g., 120% RMT). Resting or active (R/AMT, obtained with the muscle slightly contracted) MT determination is thus an important first step in the design of the rTMS protocol (Rossi et al., 2009; Lefaucheur, 2019). All the studies included in this review used this method to define their protocol. However, it can be difficult to measure the MT of the muscle to be targeted, particularly in individuals with disrupted motor pathways where MT may be very high or even absent. Researchers may then choose another **muscle for MT measurement** (in 15 studies, Table 1, column 9) which may lead to less optimal selection of stimulation intensity. Indeed, this procedure, although convenient for dealing with the MT problem, can result in insufficient (or too high) excitation of the CS specific projections of the targeted muscle leading to an absence or over-estimation of the effects which can bias the results. An alternative and standardized procedure needs to be defined for cases of absent MT.

The **targeted muscle** is chosen based on the specific population studied and the therapeutic goal. The optimal current direction on the stimulating site and thus coil orientation may vary based on the specific muscle targeted (Bashir et al., 2013; Corp et al., 2020). For example, the FDI muscle seems to be best activated with postero-anterior (PA) current (Corp et al., 2020).

Different methods were used to assess the rTMS effects in SCI (Table 2), which may explain the lack of consistency of some results. **Outcome measures** must be adapted to the study goal and be able to detect subtle changes. The clinical scale and functional outcomes commonly used in SCI studies are highly reliable (AIS, UEMS, ARAT) but may be not sensitive enough to highlight the complexity of the changes in response to modulatory interventions such as rTMS. Some neurophysiological parameters have shown poorer reliability in SCI individuals due to the injury induced-change in plasticity, especially in muscles with lower MRC (Medical Research Council, Muscle Scale) grade (Sydekum et al., 2014; Potter-Baker et al., 2016). Metrics measured from UE proximal muscle were also shown to be less reliable compared to distal ones, which may be due to the smaller cortical representation of proximal compared to distal muscle (Sankarasubramanian et al., 2015) making the latter a preferable target for rTMS. The collection of TMS metrics during a slight voluntary contraction is a known option to improve reliability. The reported dissociation between the clinical and neurophysiological changes reported by some studies (Kumru et al., 2010; Kuppuswamy et al., 2011) may be explained by the complex pathophysiology of the disease or symptom, the use of subthreshold rTMS and/or specific medication (Kumru et al., 2010) and/or the poor reliability of the neurophysiological metric, associated with a decrease of statistical power. However, such dissociation may also reveal the absence of a causal relationship between the local neuro-electrophysiological changes and the observed clinical improvements; these may be also mediated by distant effects. The relationship between neurophysiological changes and functional recovery induced by rTMS should be more consistently investigated.

The **combination of rTMS with training** or **other clinical interventions** was reported in 60% of the studied included in the review. The combination approach compared to clinical intervention alone demonstrated additional clinical and neurophysiological beneficial effects (Table 3). The added value of rTMS was demonstrated when combined with conventional rehabilitation therapy (CRT), repetitive task practice (Gomes-Osman and Field-Fote, 2015), FES (Fawaz et al., 2019), robotic (Lokomat) training (Kumru et al., 2016; Calabrò et al., 2017), and body weight-supported treadmill walking training (BWSTT; Kim and Lee, 2020; Nogueira et al., 2020). By priming the motor cortex, rTMS could be responsible for increased facilitation induced by specific motor training (Gunduz et al., 2017). These results confirm the potential of rTMS as an adjunct to the SCI rehabilitation therapy.

### Participants’ Characteristics

Many individual factors may influence the rTMS modulatory response (Ridding and Ziemann, 2010; López-Alonso et al., 2014). **Age** is the most common and widely described source of variability factor, with older participants known to show reduced potential for induced plasticity changes in response to NIBS. Individuals with SCI are usually young adults and thus exhibit a greater potential for response to rTMS and NIBS in general. The **stage and severity of the disease** may also have an influence (Jetté et al., 2013; Versace et al., 2018). Subacute and incomplete SCI individuals may have an increased potential for functional recovery and may respond better to rTMS protocols in comparison to chronic and stable SCI, who may show an activity-dependent cortical and maladaptive plasticity (Eckert and Martin, 2017). Six studies out of 20 investigated subacute SCI (1 week to 6 months; Benito et al., 2012; Ji et al., 2015; Kumru et al., 2016; Sato et al., 2018; Choi et al., 2019; Zhao et al., 2020) and three included motor and sensory complete SCI (AIS A). These studies reported the feasibility and tolerability of rTMS even at early stages and in case of severe deficits.

Individuals with different medical conditions and medications respond differently to rTMS (Leung et al., 2009). The inter-individual variability in the anatomy of the motor cortex may also reflect individual differences in the circuits activated by rTMS. All these can influence the **initial brain-state**, a well-known source of variability in response to TMS/rTMS (Bergmann, 2018). Indeed, it was observed that extreme baseline MEP values, a key factor in the TMS response, could also be partly attributable to the initial state of MEP hyperexcitability during TMS sessions (Corp et al., 2021). This initial brain state depends on the **time of the day**, the **time taken to carry out some measures**, or the **previous administration of rehabilitation therapy**.

To address the inter-individual variability issue, one option is to recruit a homogeneous **participant population** in terms of injury location, severity, and time since injury. This was often the case of the articles included in the review (Benito et al., 2012; Nardone et al., 2014a; Choi et al., 2019; Lee and Cha, 2020). To address the issue in SCI, Leszczyńska et al. (2020) reported the use of an algorithm, based on specific participant responses to single pulse TMS, to determine the rTMS parameters to use for each participant. Although the individual parameters were not explicitly reported, investigators showed a decrease in APB hand muscle spasticity associated with CSE increase. These changes were not observed for the non-targeted TA muscle.

### Conclusion and Remaining Gaps in Rtms and Sci Research

Almost all the rTMS protocols tested in SCI resulted in promising beneficial neurophysiological and/or clinical changes (Table 3). No serious side effects were reported (Table 1). Administered over several sessions (> 1week), rTMS with high number of pulses (≥1,600) administered non-continuously at subthreshold intensity and high or very-high frequency, and in combination with other rehabilitation interventions, seems appropriate to induce maintained changes in SCI. This can be explained by the cumulative plastic changes induced by repeated episodes of long-term potentiation which lead to a persistent remodeling and reorganization of the stimulated and remote areas. Future directions may extend the research field to investigate the effects of suprathreshold intensity and/or low frequency rTMS and in subacute SCI individuals. The more systematic use of neuronavigation and reporting of hotspot coordinates and rTMS induced electric fields during treatment may help increase the understanding and reproducibility of the effects observed.

A complete and detailed description of the used rTMS protocols is important. Progress has been made since the emergence of this study field and TMS experts continue to provide useful recommendations to improve reporting and ultimately designing of more effective rTMS interventions (Rodger et al., 2016; Corp et al., 2020, 2021; Lefaucheur et al., 2020; Rossi et al., 2020). However, some parameters such as the duration of the session, the number of pulses, the pulse waveform, the time of day at which sessions were administered, and the level of participant attention, have not been systematically reported in SCI studies. All these are known to influence the stimulated circuits (Di Lazzaro et al., 2001; Corp et al., 2020). Only two studies reported the use of a neuronavigation system despite its importance especially during repeated-sessions designs (Bashir et al., 2011). This may be due to the high cost of the currently available systems. Some easy-to-use and costless alternatives have been proposed and seem to provide reliable results (Cincotta et al., 2010; WashaBaugh and Krishnan, 2016; Rodseth et al., 2017; Ambrosini et al., 2018). These systems can help the systematic reporting of hotspot coordinates and monitor coil shifts across the session (Corp et al., 2020; Corp et al., 2021).

The heterogeneity of outcome measures, the lack of RCTs and the inconsistent reporting of data and statistics (means and SDs) prevented us from performing a meta-analysis. Among all the reviewed studies, no publication reported negative or complete absence of rTMS effects. Even if this is encouraging and may be explained by the use of high-frequency designs, it may also indicate publication bias (Moher et al., 2009). Despite the importance of reproducibility studies, the reporting of negative or null results could help avoid multiplication of unnecessary studies and improvement of current protocols (Bespalov et al., 2019). Explicitly specifying primary and secondary outcomes may avoid outcome reporting bias with for examples elective outcome reporting (Moher et al., 2009).

The “one-fits-all” approach in the design of rehabilitation interventions is a disputed concept, especially in the NIBS field. Some TMS stimulation parameters may need to be individually tailored based on clinical or neurophysiological state. This idea is not new (Maeda et al., 2000) and was successfully tested in one SCI study (Leszczyńska et al., 2020). The addition of neuroimaging outcomes may also be useful. A recent study reported significantly improved clinical response to rTMS when depressive patients were treated closer to personalized connectivity-guided targets (Cash et al., 2021). At the end, the stratification of the individuals in the rTMS studies could help the selection of SCI individuals more likely to respond to specific rTMS interventions.

Overall, rTMS is non-invasive, relatively easy to administer and well-tolerated intervention with promising beneficial effects on functional recovery after SCI. It is safe, with very rare to no serious side effects (Table 1; Rossi et al., 2020) and is ultimately easy to implement in clinical practice. Newly designed protocols need safety and tolerability studies, especially in the vulnerable SCI population. The best timing, intervention duration and dosage need to be clarified depending on the stage and severity of the injury. Future investigations may also focus on developing strategies to design individually-targeted rTMS interventions.

## Author Contributions

NB performed the literature review and wrote the manuscript. DA and GF were involved in the discussion of the findings and provided critical revisions. SS, GY, and JZ provided critical revisions of the final manuscript. All authors contributed to the article and approved the submitted version.

## Conflict of Interest

The authors declare that the research was conducted in the absence of any commercial or financial relationships that could be construed as a potential conflict of interest.

## Publisher’s Note

All claims expressed in this article are solely those of the authors and do not necessarily represent those of their affiliated organizations, or those of the publisher, the editors and the reviewers. Any product that may be evaluated in this article, or claim that may be made by its manufacturer, is not guaranteed or endorsed by the publisher.
